# Trends in Incidence and Transmission Patterns of COVID-19 in Valencia, Spain

**DOI:** 10.1001/jamanetworkopen.2021.13818

**Published:** 2021-06-18

**Authors:** Carolina Romero García, Adina Iftimi, Álvaro Briz-Redón, Massimiliano Zanin, Maria Otero, Mayte Ballester, José de Andrés, Giovanni Landoni, Dolores de las Marinas, Juan Carlos Catalá Bauset, Jesus Mandingorra, José Conca, Juan Correcher, Carolina Ferrer, Manuel Lozano

**Affiliations:** 1 Department of Anesthesia, Critical Care and Pain Unit, University General Hospital, Valencia, Spain; 2 Division of Research Methodology, European University, Valencia, Spain; 3 Department of Statistics and Operations Research, University of Valencia, Valencia, Spain; 4 Statistics Office, City Council of Valencia, Valencia, Spain; 5 Instituto de Física Interdisciplinar y Sistemas Complejos (CSIC-UIB), Palma de Mallorca, Spain; 6 Anesthesia Unit, Department of Surgical Specialties, Valencia University Medical School, Valencia, Spain; 7 Department of Anesthesia and Intensive Care, Istituto di Ricovero e Cura a Carattere Scientifico (IRCCS), San Raffaele Scientific Institute, Milan, Italy; 8 Faculty of Medicine, Vita-Salute San Raffaele University, Milan, Italy; 9 Division of Allergy and Immunology, University General Hospital, Valencia, Spain; 10 Department of Information Technology, University General Hospital, Universidad Católica de Valencia, Valencia, Spain; 11 Universidad Católica de Valencia. Valencia, Spain; 12 Preventive Medicine and Public Health, Food Sciences, Toxicology and Forensic Medicine Department, Universitat de Valencia, Valencia, Spain; 13 Epidemiology and Environmental Health Joint Research Unit, Foundation for the Promotion of Health and Biomedical Research of Valencia Region, Universitat Jaume I−Universitat de Valencia, Valencia, Spain

## Abstract

**Importance:**

Limited information on the transmission and dynamics of SARS-CoV-2 at the city scale is available.

**Objective:**

To describe the local spread of SARS-CoV-2 in Valencia, Spain.

**Design, Setting, and Participants:**

This single-center epidemiological cohort study of patients with SARS-CoV-2 was performed at University General Hospital in Valencia (population in the hospital catchment area, 364 000), a tertiary hospital. The study included all consecutive patients with COVID-19 isolated at home from the start of the COVID-19 pandemic on February 19 until August 31, 2020.

**Exposures:**

Cases of SARS-CoV-2 infection confirmed by the presence of IgM antibodies or a positive polymerase chain reaction test result on a nasopharyngeal swab were included. Cases in which patients with negative laboratory results met diagnostic and clinical criteria were also included.

**Main Outcomes and Measures:**

The primary outcome was the characterization of dissemination patterns and connections among the 20 neighborhoods of Valencia during the outbreak. To recreate the transmission network, the inbound and outbound connections were studied for each region, and the relative risk of infection was estimated.

**Results:**

In total, 2646 patients were included in the analysis. The mean (SD) age was 45.3 (22.5) years; 1203 (46%) were male and 1442 (54%) were female (data were missing for 1); and the overall mortality was 3.7%. The incidence of SARS-CoV-2 cases was higher in neighborhoods with higher household income (β_2_ [for mean income per household] = 0.197; 95% CI, 0.057-0.351) and greater population density (β_1_ [inhabitants per km^2^] = 0.228; 95% CI, 0.085-0.387). Correlations with meteorological variables were not statistically significant. Neighborhood 3, where the hospital and testing facility were located, had the most outbound connections (14). A large residential complex close to the city (neighborhood 20) had the fewest connections (0 outbound and 2 inbound). Five geographically unconnected neighborhoods were of strategic importance in disrupting the transmission network.

**Conclusions and Relevance:**

This study of local dissemination of SARS-COV-2 revealed nonevident transmission patterns between geographically unconnected areas. The results suggest that tailor-made containment measures could reduce transmission and that hospitals, including testing facilities, play a crucial role in disease transmission. Consequently, the local dynamics of SARS-CoV-2 spread might inform the strategic lockdown of specific neighborhoods to stop the contagion and avoid a citywide lockdown.

## Key Points

**Question:**

How does SARS-CoV-2 spread within a city, and are there instrumental neighborhoods that might modulate the spread?

**Findings:**

This epidemiological cohort study of 2646 patients with COVID-19 conducted in the third most populated city in Spain found that the neighborhood where the COVID-19 testing facility was located also had the highest number of total connections (both inbound and outbound). The mean income and population density had a direct correlation with the number of cases.

**Meaning:**

These findings suggest that a selective and strategic lockdown of specific neighborhoods could help reduce the spread of SARS-CoV-2.

## Teaser

This epidemiological cohort study describes the local transmission pattern of SARS-CoV-2 in Valencia, Spain.

## Introduction

Some studies have described SARS-CoV-2 transmission using online public data,^1,2,3,4,5^ but limited information describes local COVID-19 transmission. Changes in mobility,^6^ social distancing measures,^7^ and quarantine and lockdown effects^1^ influence the spread of SARS-CoV-2. In addition, meteorological conditions, pollution, and economic factors might play a nonnegligible role in virus spread.^8^ Full lockdown is a strategy that has been widely used to flatten the curve and reduce the spread of COVID-19.^9^

The first confirmed case of COVID-19 in Valencia, Spain, was reported on February 19, 2020; a citywide lockdown began on March 14, 2020, and lasted until May 15, 2020. The city’s infection rate followed a heterogeneous distribution during the outbreak, and our institution, the University General Hospital, was designated as a COVID-19 center.

Neighborhoods constitute the core elements and operating units in any public health response. Knowledge of the spatial distribution and geographical characteristics is paramount to plan infection control measures and address local outbreaks. Thus, this study aimed to define the dynamics of SARS-CoV-2 transmission by targeting specific regions in the city of Valencia. Herein, we present a city-scale study in a tertiary hospital that serves approximately 364 000 people in the catchment area. We examined the local dissemination of SARS-CoV-2 during the first 6 months of the pandemic, considering the first sequentially diagnosed patients in 20 neighborhoods of the city.

## Methods

The study was conducted at the University General Hospital, an academic public hospital that serves the largest area in Valencia. The study was approved by the institutional review board of the hospital, and the requirement for informed consent was waived. We adhered to the Strengthening the Reporting of Observational Studies in Epidemiology (STROBE) reporting guideline for cohort studies.^10^

From February 19 to August 31, 2020, we recruited all consecutive patients who had positive test results for COVID-19 and an Ordinal Scale for Clinical Improvement score^11^ from 0 to 2 (range, 0-6, with higher scores indicating worse outcomes). To analyze the space-time evolution of the pandemic and the interactions among regions in the study area, patients’ home addresses were retrieved from medical records according to the local census bureau. Overall, 3643 addresses were converted into geographic coordinates (longitude and latitude) through automatic assignment (using the Spanish CartoCiudad mapping system^12^) and were manually revised/corrected by specialized personnel. During this process, 132 patients were excluded because of technical problems in assigning coordinates or the absence of coordinates for their addresses. Another 799 patients were also excluded because they were residents of neighborhoods that are not part of the study area. A total of 66 patients directly admitted to the intensive care unit were also excluded. Therefore, 2646 patients were included in the analysis. The study area comprised all 20 neighborhoods located within the hospital’s catchment area. For simplicity, Roman numerals were used to rename the 20 areas under study; the number I was used to identify the city center. Larger numbers indicate neighborhoods more distant from the city center. The first case was recorded on February 19, 2020, in neighborhood 14. The onset of the disease was defined as the first day of follow-up. Afterward, a confirmatory test was prescribed: either the polymerase chain reaction test in a nasopharyngeal swab or a serological test for the presence of viral antibodies (IgM).

### Statistical Analysis

To evaluate the spatiotemporal evolution of the pandemic in the study area, we adjusted a Knorr-Held^13^ model. This model combines the spatial model by Besag et al^14^ with the temporal and spatiotemporal random effects that allow the combination of nonseparable effects in space and time. To assess COVID-19 propagation among the 20 neighborhoods, we applied the Granger causality test,^15^ a test designed to detect associations between pairs of time series beyond simple correlations. A more concise explanation of the spatial model and the propagation networks is presented in eMethods in the Supplement. eFigure 1 in the Supplement shows a flowchart of the statistical analysis plan. Statistical analysis was performed in R, version 3.3.^16^ The integrated nested Laplace approximations (INLA) package^17^ was used to reduce computation time, providing bayesian inference by means of the user-friendly library R-INLA.^18^ Functional network analyses were performed in Python, version 3.8.^19^ The Granger causality test is described in StatsModels library,^20^ whereas network metrics were extracted using the NetworkX library.^21^ Two-sided *P* < .05 indicated statistical significance.

## Results

Among the 2646 patients included in the analysis, the mean (SD) age was 45.3 (22.5) years, 1203 (46%) were male, and 1442 (54%) were female (data were missing for 1). The overall mortality was 3.7%. The mean (SD) time from diagnosis to death was 41.2 (33.7) days.

**Figure 1.**
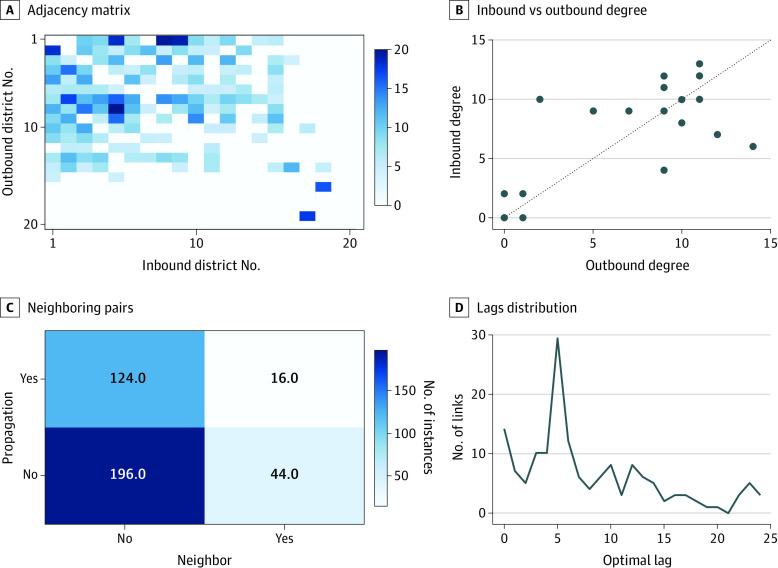
Connectivity Structure From Functional Networks Analysis A, Resulting adjacency matrix. White cells indicate the absence of a propagation process, whereas darker shades indicate its strength. Neighborhoods are ordered as in the Table. B, Inbound degree of nodes as a function of their outbound degree. The dashed gray line represents the main diagonal. C, Distribution of the number of pairs of neighborhoods as a function of being neighbors and the presence of propagation between them. D, Distribution of the number of propagation links as a function of their optimal time lag.

Figure 1 shows the connectivity structure resulting from functional network analyses. Darker cells show stronger propagation dynamics for SARS-CoV-2 transmission (ie, larger values of weight matrix and smaller values of the Granger test *P* value) (Figure 1A), indicating that most neighborhoods were embedded in a densely connected structure, suggesting easy propagation of COVID-19 between separate areas in the city. Nevertheless, 5 neighborhoods were more isolated than the rest, and neighborhood 20 reported no cases. Figure 1B presents a scatterplot of the in-degree of each node as a function of their out-degree. Although both metrics were partly correlated (Pearson *r*^2^ = 0.658), the presence of points far away from the diagonal suggests that disease propagation was not a strictly symmetrical process.

To further characterize this propagation structure, Figure 1C presents a contingency table in which all possible neighborhood pairs were characterized by (1) whether they were spatial neighbors and (2) whether a propagation link was detected between them by the Granger test. Although a χ^2^ test cannot reject the hypothesis of independence (*P* = .08), the large *P* value suggests a propagation dynamic mostly free from geographical limitations. Finally, Figure 1D shows the distribution of the optimal lags (ie, of those time lags minimizing the *P* value of the Granger causality test, or of how fast the disease propagated between 2 neighborhoods). The optimal lag obtained was 5 days in the propagation network.

The Table shows the role of each neighborhood in the propagation process and the ranking according to the total degree value (ie, the total number of connections). The *z* score of the degree value (in parentheses) was calculated by comparing the observed value with the expected value when the time series of each neighborhood was randomly shuffled. This value thus represents how unexpected the observed degree is, when compared with a situation in which no association between neighborhoods is present, and is thus a proxy of its statistical significance. The neighborhood with the highest number of outbound connections was neighborhood 3. The number of outbound links was 14, and the number of inbound links was only 6. In contrast, neighborhood 7 showed the opposite behavior, with a low number of inbound connections (2) and a high number of outbound ones (10). The ranking of the top 5 neighborhoods according to their normalized betweenness centrality value was as follows: neighborhood 10 had the highest betweenness centrality of 1.00, followed by neighborhood 9 with 0.88, neighborhood 13 with 0.74, neighborhood 1 with 0.69, and neighborhood 6 with 0.59.

**Table.** Ranking of Study Neighborhoods According to Total Degree of Connections^a^

Rank	Neighborhood	Degree value (*z* score)		
		Total	Outbound	Inbound
1	10	24 (30.49)	11 (20.54)	13 (22.91)
2	6	23 (31.77)	11 (22.33)	12 (21.54)
3	9	21 (26.37)	9 (15.45)	12 (20.95)
4	8	21 (26.35)	11 (20.67)	10 (16.55)
5	18	20 (14.36)	9 (8.54)	11 (11.37)
6	2	20 (35.26)	10 (26.41)	10 (23.79)
7	3	20 (34.59)	14 (34.21)	6 (14.66)
8	11	19 (28.65)	12 (24.32)	7 (15.69)
9	12	18 (19.37)	10 (15.50)	8 (11.82)
10	13	18 (15.71)	9 (11.46)	9 (10.83)
11	15	16 (25.17)	7 (15.85)	9 (19.72)
12	16	14 (14.36)	5 (6.55)	9 (13.63)
13	4	13 (37.05)	9 (36.86)	4 (15.68)
14	1	13 (17.69)	9 (17.02)	4 (7.36)
15	7	12 (15.40)	2 (3.06)	10 (18.51)
16	14	3 (1.61)	1 (0.50)	2 (1.78)
17	20	2 (–0.57)	0 (–1.23)	2 (0.49)
18	5	2 (0.03)	0 (–1.04)	2 (1.10)
19	17	1 (–0.20)	1 (0.51)	0 (–0.81)
20	19	0 (NA)	0 (NA)	0 (NA)

Abbreviation: NA, not applicable.

^a^ The total degree of connections was defined as the sum of the number of outbound or departing connections and inbound or arriving connections.

**Figure 2.**
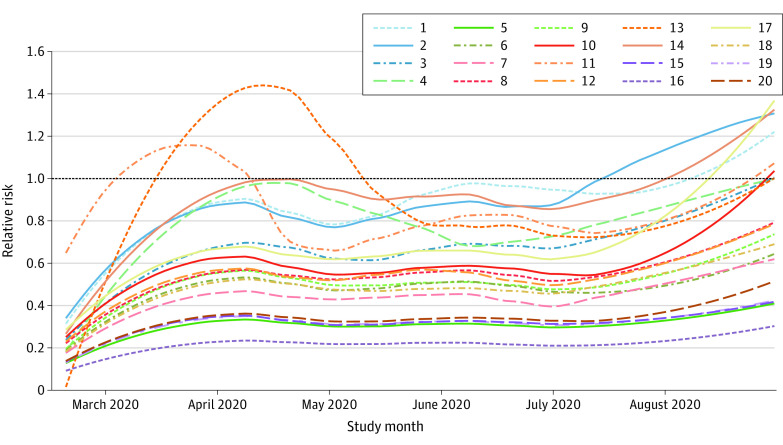
Smoothed Relative Risks Estimation The locally estimated risk was obtained using a locally estimated scatterplot smoothing regression for each neighborhood (1-20).

The evolution of relative risks for the 20 neighborhoods is shown in Figure 2. The daily relative risks have been smoothed using a locally estimated scatterplot smoothing regression technique.^22^ A general temporal evolution could be assessed for most of the relative risk curves in each neighborhood: (1) relative risks remained low until March; (2) they grew until mid-April; (3) they decreased and remained nearly constant for the following 2 months; and (4) they started to grow again from mid-July. In addition to this general behavior, which varied moderately by neighborhood, some trajectories were noteworthy, particularly those that corresponded to neighborhoods 11, 13, and 2 (Figure 2). In addition, neighborhood 13 reached the highest values of smoothed relative risk among all areas and was associated with the first wave of the pandemic. Moreover, neighborhoods 13 and 3 did not show such a high relative risk at any time during the study period. Although neighborhood 13 was mainly followed by the high risk experienced by neighborhood 11, the risk growth in neighborhood 2 was parallel with that in several other neighborhoods.

**Figure 3.**
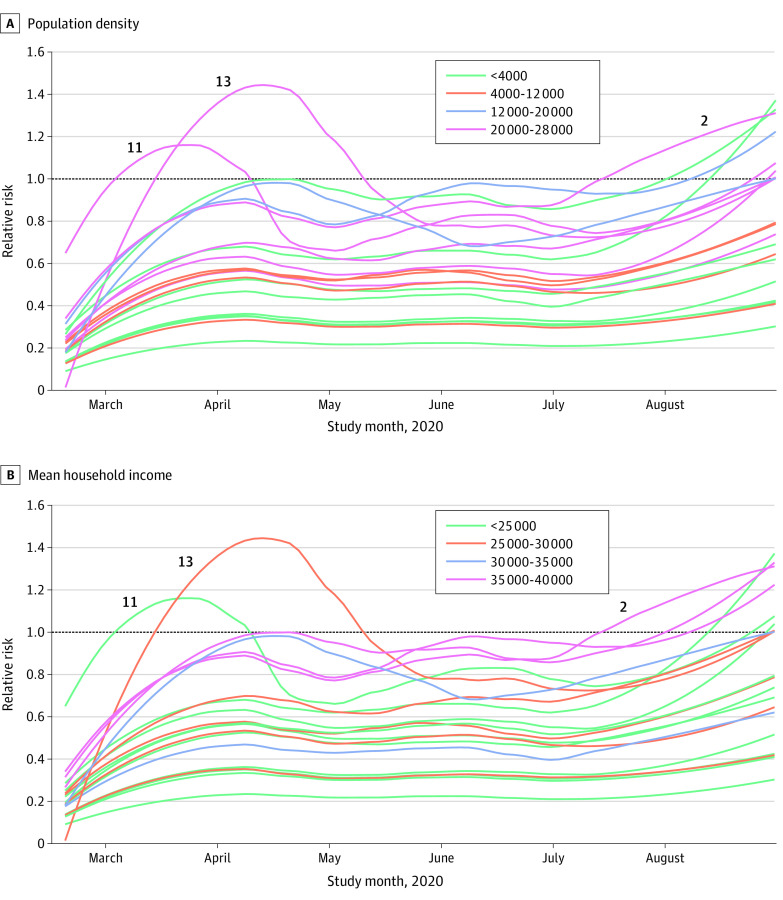
Evolution of Smoothed Relative Risks by Neighborhood Evolution was measured using a locally estimated scatterplot smoothing regression of each neighborhood. Population density is measured in inhabitants per square kilometer; mean household income, in euros.

The trends in each neighborhood since the start of the pandemic are shown in Figure 3. Both the population density (β_1_ [inhabitants per km^2^] = 0.228; 95% CI, 0.085-0.387) and mean household income (β_2_ [mean income per household] = 0.197; 95% CI, 0.057-0.351) showed statistically significant differences. In contrast, there was no association between relative risk estimates and the 3 meteorological covariates, temperature (β_3_ = –0.053; 95% CI, –0.289 to 0.183), mean wind speed (β_4_ = 0.026; 95% CI, –0.042 to 0.092), and hours of sunlight (β_5_ = –0.010; 95% CI, –0.089 to 0.068).

**Figure 4.**
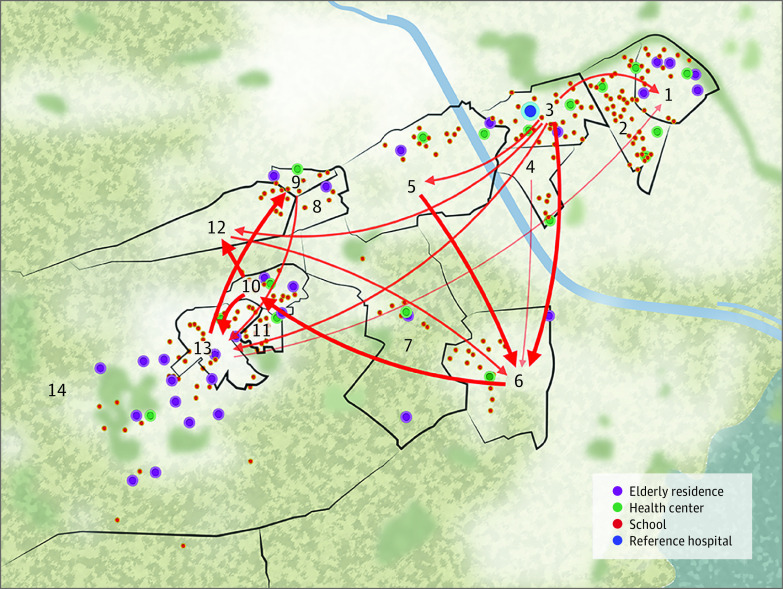
Functional Propagation Network and Interactions Among Neighborhoods The thickness of an arrow indicates the strength of the propagation link; thicker arrows represent stronger links, whereas thinner arrows represent weaker links.

Figure 4 shows the network reconstruction, including schools, nursing homes, and state-run health clinics, from the region of interest. The spatiotemporal representations of the incidence rates and relative risks are presented in eFigure 2 in the Supplement.

## Discussion

To our knowledge, this is the first large city-scale epidemiological study of SARS-CoV-2 transmission and the first work proposing a functional network approach to model the local propagation of an infectious disease. We investigated the temporal dynamics of the SARS-CoV-2 outbreak using a city-scale model. The study included patients with COVID-19 discharged to self-isolation at home from a tertiary hospital. Several studies at a national scale have investigated the transmission and propagation of COVID-19^23,24^ and modeled quantitative information of positive cases, deaths, and intensive care unit beds,^4,25,26,27^ but limited information is available on the transmission patterns within a city.

The association between population density and number of COVID-19 cases has been described extensively,^28^ confirming our results. Meanwhile, the role of climate on the dissemination of the virus remains unclear. Some studies did not find an association,^29,30,31^ whereas others suggest that lower temperature and humidity conditions favored transmission.^32^ Although no influence of climate was observed in the present study, further epidemiological studies with different meteorological conditions might shed more light on this. In addition, the positive association between COVID-19 cases and mean household income in our study contradicts previous results.^33^ One possible explanation for this is the fact that many of the neighborhoods in this study were located in different municipalities. Thus, some neighborhoods with a lower mean income were, simultaneously, among the most isolated in terms of transportation, commercial, and industrial activities, which are potential confounders of the association between COVID-19 risk and the income level. Furthermore, the association between income level and COVID-19 risk could vary enormously between countries, depending on factors such as health care accessibility in economic and geographical terms.

The results of the present study suggest that mobility data are not an essential prerequisite for the study of the spreading process, but that complex propagation patterns can instead be described by means of functional networks reconstructed on top of local data alone. In our case, this allowed us to highlight several interesting patterns: (1) different parts of the city were heterogeneous in their degree of connectivity, with some neighborhoods being virtually isolated; (2) the propagation of COVID-19 could be highly asymmetric, with some neighborhoods being net receivers (sinks) and others net propagators (sources); and (3) geographical proximity was not the main factor driving propagation, suggesting the predominance of long-range mobility patterns.

Our findings suggest significant variability across neighborhoods. Several neighborhoods had a degree value substantially higher than that expected in the random case, suggesting a robust propagation process. Clusters of cases disseminate within a city with a lag of 5 days.^23,34^ Therefore, it seems that an effective action plan designed to control spread should be implemented within a period no longer than 5 days after the first case is detected.

Neighborhood 7, a large family residential complex close to the city, had received many connections from other areas but did not contribute to disease propagation. That is, people in this area had acquired the disease in the city during the lockdown. The opposite occurred in the area in which the hospital was located. Neighborhood 3 had the most outbound links and received few propagation connections from other regions. The persistence of mobility during the lockdown would explain this result, but such a conclusion cannot be derived from available data alone. Thus, a future prospective analysis with a higher cumulative incidence of cases might provide more accurate results.

In the analysis of betweenness, neighborhoods 13 and 1 showed high centrality, although they ranked low in the total degree (in positions 10 and 14, respectively). Neighborhood 13 was the entrance of a large municipality that connected this village with the city. Another relevant area was neighborhood 1, the most centered neighborhood in the city. The betweenness centrality metric is designed to assess how much a propagation process would be hindered by the deletion of a node or, conversely, how essential a node is in the propagation of information throughout a network. Therefore, and despite their low connectivity with other neighborhoods in the area, the high centrality of these neighborhoods suggests that they may have been instrumental in the global propagation of COVID-19, at least from a Granger viewpoint, and that they may therefore be good candidates for selective containment measures.

Overall, these results seem to depict a complex propagation process, in which neighborhoods are strongly connected but in an uneven manner, partly independent of geographical locations. However, some areas seemed to escape this process and maintained notable isolation.

### Limitations

This study has several limitations. First, only 1 hospital in Valencia was studied. Second, more than 800 patients were excluded because of a mismatch in their permanent address. Third, the absence of specific data on mobility patterns or transportation methods might have biased the association between the household income and the number of COVID-19 cases. Fourth, during the outbreak, the indications for SARS-CoV-2 testing changed with time. Fifth, the transmission patterns of COVID-19 in a single center within a city would need external validation. Finally, the linear nature of the Granger causality test needs to be considered.

## Conclusions

In this cohort study, functional network analyses revealed geographic regions that are not interconnected but have a nonevident mobility pattern that influences SARS-CoV-2 transmission. The major observation was that hospitals with a testing facility might become a major contributor to local spread. The relocation of testing sites to isolated areas could represent a containment measure per se, but this hypothesis remains unproven. Evidence on the embeddedness and connectedness of districts in a city exists, and tailor-made lockdowns could be designed to control local outbreaks.

## Notes

Supplement. **eMethods.** Statistical Analysis Protocol eReferences **eFigure 1.** Data Analysis Plan **eFigure 2.** Spatiotemporal Representation of Relative Risks and Incidence Rate
