# Supplementary material for: Trends in Incidence and Transmission Patterns of COVID-19 in Valencia, Spain
Source: JAMA Netw Open. 2021 Jun 18;4(6):e2113818. doi: 10.1001/jamanetworkopen.2021.13818 (PMC8214162; doi:10.1001/jamanetworkopen.2021.13818)
Supplement: Supplement. — eMethods. Statistical Analysis Protocol eReferences eFigure 1. Data Analysis Plan eFigure 2. Spatiotemporal Representation of Relative Risks and Incidence Rate [file jamanetwopen-e2113818-s001.pdf]

Supplementary Online Content

Romero García C, Iftimi A, Briz-Redón Á, et al. Trends in incidence and transmission patterns of COVID-19 in Valencia, Spain. *JAMA Netw Open*. 2021;4(6):e2113818. doi:10.1001/jamanetworkopen.2021.13818

eMethods. Statistical Analysis Protocol
eReferences
eFigure 1. Data Analysis Plan
eFigure 2. Spatiotemporal Representation of Relative Risks and Incidence Rate

This supplementary material has been provided by the authors to give readers additional information about their work.

© 2021 Romero García C et al. *JAMA Network Open*.

eMethods. Statistical Analysis Plan

Spatial Model

In the Besag[1] model the risk associated with a region is modelled as the sum of a heterogeneity and a clustering effect. For the temporal effect we want a smooth and flexible evolution, thus we consider a structured random effect in order to make sure that periods close in time are expected to be similar and also allowing for flexible forms for the temporal evolution curves.

For this study, we assumed O_{ij} to be the number of observed cases and E_{ij} the expected number of cases for the *i*th neighborhood and *j*th day within the period. Hence, the number of observed cases was modelled as $O_{ij} \sim$ Poisson (r_{ij} E_{ij}), where r_{ij} is the underlying relative risk for COVID-19 infection. The relative risk represents the ratio between the number of estimated cases provided by the model and the expected number of cases in i*th* neighborhood on day j*th*. E_{ij} was calculated as the total number of cases observed on day *j* multiplied by the fraction of the population that neighborhood *i* represents. The following equation shows the general definition of the additive spatiotemporal model:

$$log(r_{ij}) = \alpha + \sum_{k=1}^{n} \beta_k x_{kij} + \theta_i + \phi_i + \rho_j + \gamma_j + \delta_{ij}, \quad \text{Eq. (1)}$$

where α is the global intercept of the model; $\beta_k's$ represent covariate (x_k) effects; θ_i and ϕ_i are two spatial effects adopting the standard model[2] with structured and unstructured components, respectively; and ρ_j and γ_j represent the structured and unstructured temporal effects. A Gaussian Conditionally Autoregressive (CAR)

distribution was chosen to model the spatially-structured random effect. The usual contiguity matrix which considers that two areas are neighbors if they share a geographical border was considered to define this prior. An independent zero-mean normal prior was used for the temporal effect γ. In contrast, the parameter ρ displays a temporal structure. We considered a second-order random walk (RW2) for ρ, with a prior in which effects for neighboring time points tend to be alike. The parameter δ represents space-time interaction. The spatio-temporal interaction term was modeled through a Gaussian prior, which corresponds to the type I interaction in the context of the Knorr-Held models. Specific constraints on the random effects have been considered to avoid identifiability issues.[3]

The following covariates were studied: population density (x_1, in inhabitants/km^2); average income per household (x_2, in euro); and three meteorological variables, namely average temperature (x_3, in °C), average wind speed (x_4, in km/h), and number of sunlight hours (x_5, number of hours in which solar irradiance is >120 W/m^2). Population density and income data were obtained from the National Statistics Institute. The effect of the meteorological covariates was considered on a daily basis and with a 7-day lag. An ordinary kriging model[4] was used to estimate the covariate values. Daily measurements were collected from the meteorological stations of the National Weather Service (AEMET).

Functional Networks

Propagation Assessment

© 2021 Romero García C et al. *JAMA Network Open*.

The Granger´s causality test[5] is based on two intuitive concepts: causes must precede the corresponding effects and the prediction of the caused time series should be improved when using information from the causes. It has been extensively applied to economic[6] and biomedical data.[7, 8] If the propagation of COVID-19 occurs from neighborhood B to neighborhood A, a forecast of the number of cases in A should be more precise when information from the past of B is also included. Reversing the argument, such forecast can then be used to infer the presence of a propagation process, without the need of a priori information as mobility data, but only relying on the local evolution of the pandemics.

Considering the time series of the number of new cases in two neighborhoods A and B, it is said that B “Granger-causes” A if:

$$\sigma^2(A|U^-) < \sigma^2(A|U^- \backslash B^-), \qquad \text{Eq. (2)}$$

where $\sigma^2(A|U^-)$ is the error (standard deviation of residuals) in forecasting the time series A, using past information of the entire universe U, and $\sigma^2(A|U^- \backslash B^-)$ is the error when the time series B is not considered. Once again, B is said to cause A if including information about the past of B reduces the error in the forecast of the future of A. Even if the test has the word “causality” in its name, this is not a true causality in the sense of a randomized trial; as such, instances of statistically significant Granger tests will here be called associations.

The Granger test has been applied to the time series representing the evolution of the number of cases through time for each pair of neighborhoods; thus, the result indicates whether an association between the evolution of two neighborhoods is

© 2021 Romero García C et al. *JAMA Network Open*.

present. Before applying it, time series have been checked for stationarity, i.e. for the absence of linear and periodic trends; note that the presence of such trends is known to lead to spurious results in a Granger test. Stationarity has been assessed using an augmented Dickey-Fuller unit root test.[9] While half of them, the presence of a unit root could not be discarded (for $\alpha = 0.01$), an autocorrelation analysis showed that this was mainly due to a periodicity of 7 days. This, nevertheless, did not affect the results, as most of the causality relationships were found for shorter time lags (Fig. 1a). The associations detected by the Granger test were on a shorter time scale than the trends, thus the impact of the latter ones is negligible. The forecast of the time series was then performed through an autoregressive-moving-average model,[10] in which the time series of the causing element was shifted a number of time steps back in time. An F-test was finally applied to assess the statistical significance of this inequality of Eq (2) and to obtain a corresponding *p*-value.

Propagation Network Reconstruction

The complete COVID-19 propagation network was reconstructed by considering all possible pairs of neighborhoods (i, j), with $i \neq j$, and by calculating the Granger causality between the corresponding time series. Tests whose *p*-values were above the statistical significance threshold ($[\alpha = 0.01]$, with Bonferroni correction for multiple comparisons) were discarded. An adjacency matrix $\boldsymbol{A}$ of size $n \times n$ was then created, with $n = 20$ being the number of neighborhoods, such that the element $a_{i,j} = 1$ if a temporal association was accepted between neighborhoods i and j, and $a_{i,j} = 0$ otherwise.[11,12] This kind of network is known in statistical physics as a 'functional

© 2021 Romero García C et al. *JAMA Network Open*.

network', in which the dynamics of nodes is expected to be a *function* of the connectivity; the latter (the unknown part) is then reconstructed through the former (the known data).[13] We further calculated a weight matrix $\boldsymbol{W}$, encoding how strong the propagation was between pairs of neighborhoods. For each pair (i, j) with $a_{i,j} = 1$, $w_{i,j}$ was defined as $-\, log_{10}\, p_{i,j}$, where $p_{i,j}$ is the *p*-value obtained by the Granger test for neighborhoods i and j. Therefore, the larger the value $w_{i,j}$, the clearer the propagation process from a Granger point of view.

Note that the adjacency matrix $\boldsymbol{A}$ and the weight matrix $\boldsymbol{W}$ represent complementary views of the same information. Each element (i, j) in them is derived from the same Granger test, and specifically from the corresponding *p*-value. $a_{i,j}$ yields a binarized view of the *p*-value, i.e. whether a statistically significant association exists; on the other hand, $w_{i,j}$ indicates the strength of such association. This dual view of the connectivity structure is customary in network science, and each matrix is used to characterize different aspects of it.

The resulting network was analyzed in terms of the following metrics[14]:

- Out-degree: number of outbound links from a given node, or number of neighborhoods the vector is propagating. This value corresponds to the row-sum of the adjacency matrix $\boldsymbol{A}$.
- In-degree: number of inbound links at a given node, or number of neighborhoods propagating to it.
- Total degree: sum of inbound and outbound links of a node.

© 2021 Romero García C et al. *JAMA Network Open*.

- Betweenness centrality: how instrumental or strategical a neighborhood was in propagating the disease throughout the whole network, defined the sum of the fractions of all-pair shortest paths that pass through that node.[15] In other words, this centrality assesses how many times a neighborhood has mediated the propagation between two other regions. The distance between pairs of nodes, which corresponds to the dissimilarity between the corresponding neighborhoods, is defined as the inverse of $w_{i,j}$. The resulting values were normalized such that the most central node has a betweenness centrality of 1.

References

1. Besag J, York J, Mollié A. Bayesian image restoration, with two applications in spatial statistics. Ann Inst Stat Math. 1991;43(1):1-20. doi:10.1007/BF00116466

2. Cartociudad. datanalytics. Accessed November 5, 2020. Posted online https://www.datanalytics.com/2016/03/31/cartociudad/

3. Goicoa T, Adin A, Ugarte MD, Hodges JS. In spatio-temporal disease mapping models, identifiability constraints affect PQL and INLA results. Stoch Environ Res Risk Assess. 2018 Mar 1;32(3):749–70

4. Cressie N. Spatial prediction and ordinary kriging. Math Geol. 1988;20(4):405-421. doi:10.1007/BF00892986

5. Granger CWJ. Some recent development in a concept of causality. J Econ. 1988;39(1-2):199-211. doi:10.1016/0304-4076(88)90045-0

6. Hoover K. Causality in macroeconomics. Cambridge University Press; 2001. Accessed October 27, 2020. Posted online https://econpapers.repec.org/bookchap/cupcbooks/9780521452175.htm

© 2021 Romero García C et al. *JAMA Network Open*.

7. Bressler SL, Seth AK. Wiener-Granger causality: A well established methodology. NeuroImage. 2011;58(2):323-329. doi:10.1016/j.neuroimage.2010.02.059

8. Kleinberg S, Hripcsak G. A review of causal inference for biomedical informatics. J Biomed Inform 2011;44(6):1102-1112. doi:10.1016/j.jbi.2011.07.001

9. MacKinnon JG. Approximate asymptotic distribution functions for Unit-Root and Cointegration Tests. J Bus Econ Stat. 1994;12(2):167-176. doi:10.1080/07350015.1994.10510005

10. Brockwell PJ, Davis RA, Fienberg SE. Theory and methods | Peter J. Brockwell | Springer Time Series. Accessed October 27, 2020. Posted online https://www.springer.com/gp/book/9780387974293

11. Strogatz SH. Exploring complex networks. Nature. 2001;410(6825):268-276. doi:10.1038/35065725

12. Boccaletti S, Latora V, Moreno Y, Chavez M, Hwang D-U. Complex networks: Structure and dynamics. Phys Rep. 2006;424(4-5):175-308. doi:10.1016/j.physrep.2005.10.009

13. Zanin M, Papo D, Sousa PA, et al. Combining complex networks and data mining: Why and how. Phys Rep. 2016;635:1-44. doi:10.1016/j.physrep.2016.04.005

14. Costa LdF, Rodrigues FA, Travieso G, Villas Boas PR. Characterization of complex networks: A survey of measurements. Adv Phys. 2007;56(1):167-242. doi:10.1080/00018730601170527

15. Freeman LC. A set of measures of centrality based on betweenness. Sociometry. 1977;40(1):35-41. doi:10.2307/3033543

© 2021 Romero García C et al. *JAMA Network Open*.

eFigure 1. Data Analysis Plan

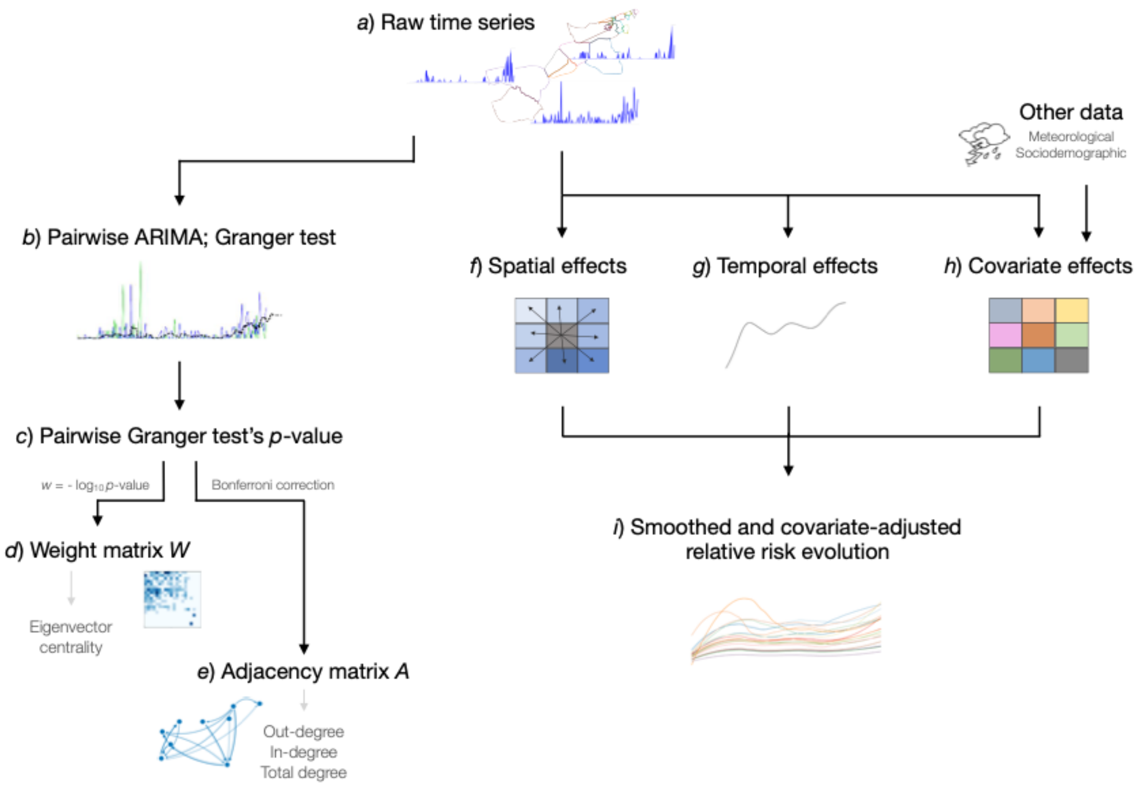

© 2021 Romero García C et al. *JAMA Network Open*.

eFigure 2. Spatiotemporal Representation of Relative Risks and Incidence Rate

The blue dot represents the University General Hospital.

A

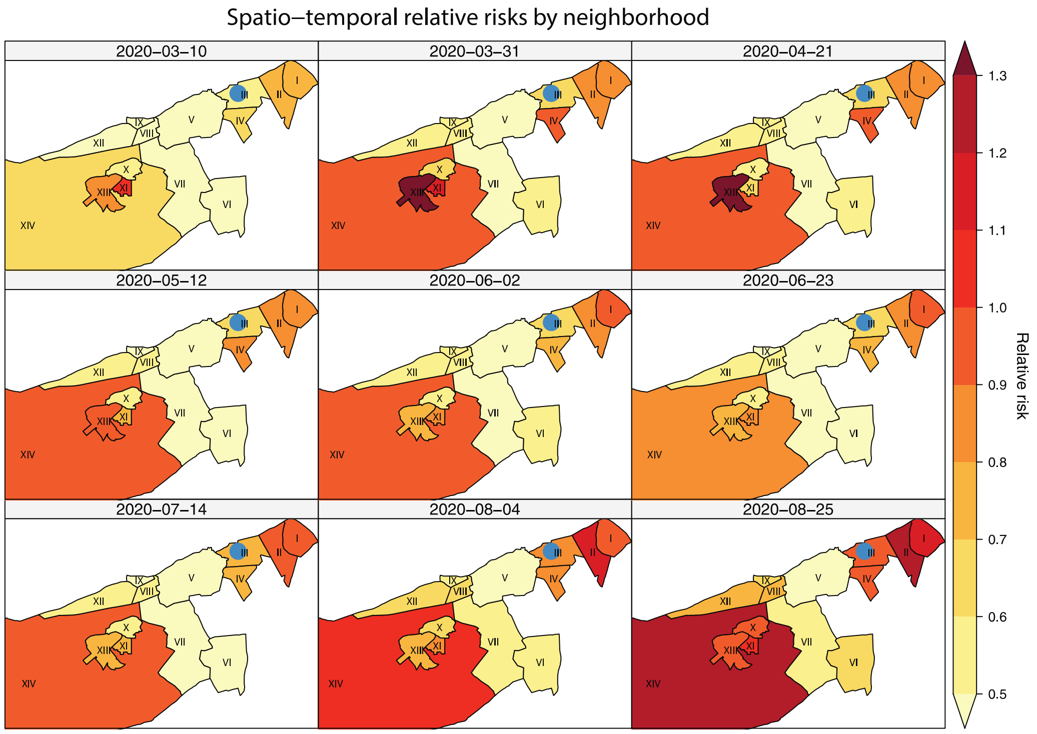

B

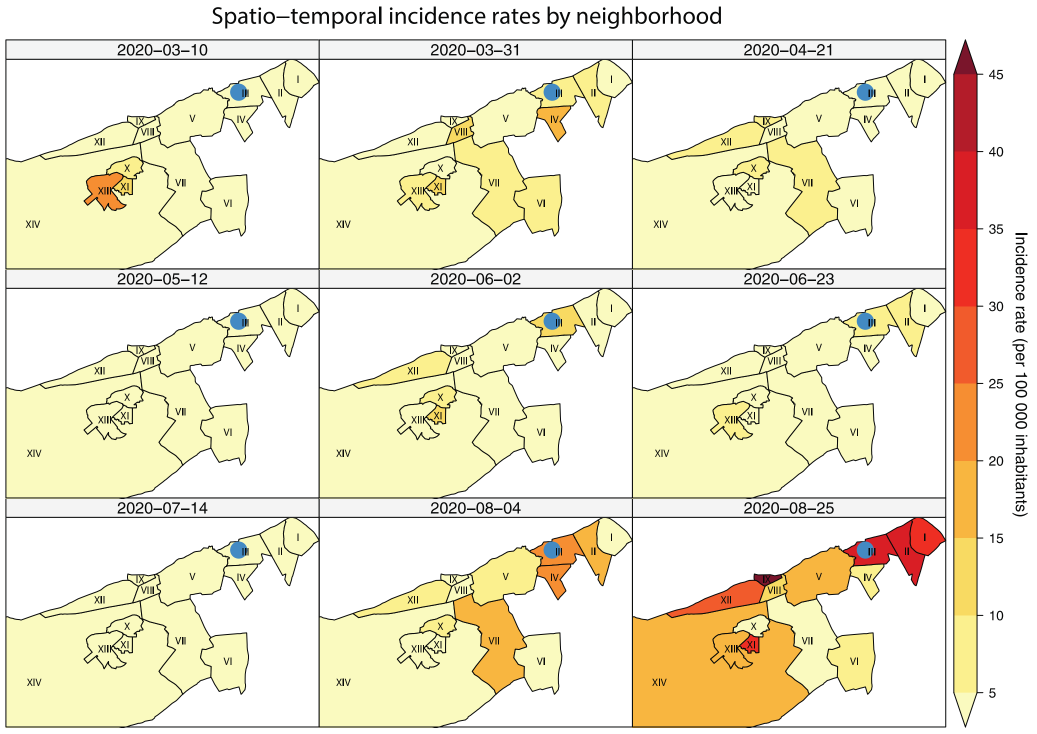

© 2021 Romero García C et al. *JAMA Network Open*.
